# Identification of regulatory cell death-related genes during MASH progression using bioinformatics analysis and machine learning strategies

**DOI:** 10.3389/fimmu.2025.1542524

**Published:** 2025-05-08

**Authors:** Zhiqiang Lin, Weiyi Li, Yuan Xu, Hangchi Liu, Yufei Zhang, Ruifen Li, Wenqian Zhao, Youfei Guan, Xiaoyan Zhang

**Affiliations:** ^1^ Health Science Center, East China Normal University, Shanghai, China; ^2^ Department of Nephrology, Affiliated Hospital of Nantong University, Medical School of Nantong University, Nantong, China; ^3^ Advanced Institute for Medical Sciences, Dalian Medical University, Dalian, China

**Keywords:** MASH, liver fibrosis, RCD, machine learning, bioinformatics

## Abstract

**Background:**

Metabolic dysfunction-associated steatohepatitis (MASH) is becoming increasingly prevalent. Regulated cell death (RCD) has emerged as a significant disease phenotype and may act as a marker for liver fibrosis. The present study aimed to investigate the regulation of RCD-related genes in MASH to elucidate the role of RCD in the progression of MASH.

**Methods:**

The gene expression profiles from the GSE130970 and GSE49541 datasets were retrieved from the Gene Expression Omnibus (GEO) database for analysis. A total of 101 combinations of 10 machine learning algorithms were employed to screen for characteristic RCD-related differentially expressed genes (DEGs) that reflect the progression of MASH. Gene Ontology (GO) and Kyoto Encyclopedia of Genes and Genomes (KEGG) analyses were conducted to explore the enrichment pathways and functions of the feature genes. we performed cell classification analysis to investigate immune cell infiltration. Consensus cluster analysis was performed to identify MASH subtypes associated with RCD. The GSE89632 dataset was utilized to analyze the correlation of characteristic genes with clinical features of MASH. The DGIdb database was employed to screen for potential therapeutic drugs and compounds targeting the feature genes. In addition, we established mouse liver fibrosis models induced by methionine-choline-deficient (MCD) diet or CCl4 treatment, and further validated the expression of characteristic genes through quantitative real-time PCR (q-PCR). Lastly, we knocked down EPHA3 in LX2 cells to explore its effect on TGFb-induced activation of LX2 cells.

**Results:**

This study discovered a total of 11 RCD-associated DEGs, which predicted the progression of MASH. Advanced MASH has higher levels of immune cell infiltration and is significantly correlated with the RCD-related DEGs expression. MASH can be classified into two subtypes, cluster 1 and cluster 2, based on these feature genes. Compared with cluster 1, cluster 2 has highly expressed RCD-related DEGs, shows an increase in the degree of fibrosis. Furthermore, We discovered that the expression levels of feature genes were positively correlated with AST and ALT levels. Subsequently, We also evaluated the expression of these 11 feature genes in the liver tissues of mice with fibrosis induced by MCD or CCl4, and the results suggested that these genes may be involved in the development of fibrosis. WB results showed that the protein level of EPHA3 significantly increased in both mouse models of liver fibrosis. *In vitro*, we observed that knocking down EPHA3 in LX2 cells significantly inhibited the activation of the TGF-β/Smad3 signaling pathway.

**Conclusion:**

Our study sheds light on the fact that RCD contribute to the progression of MASH, high lighting potential therapeutic targets for treating this disease.

## Introduction

Non-alcoholic fatty liver disease (NAFLD) is one of the most prevalent chronic liver diseases worldwide and poses a significant threat to public health (1). The disease has now been renamed metabolic dysfunction-associated fatty liver disease (MAFLD) (2). The initial stage in the development of MAFLD is simple hepatic steatosis, which is characterized by the accumulation of large triglyceride vacuoles within hepatocytes. Diagnosis is based on imaging or histological examination that reveals steatosis in at least 5% of liver cells, along with the exclusion of excessive alcohol consumption or other concurrent causes of liver disease (3). MASH represents a more severe form of MAFLD. The diagnosis of MASH requires confirmation of steatosis, lobular inflammatory infiltrates, hepatocellular ballooning, and fibrosis through liver biopsy (4). Up to 30% of patients with MAFLD may progress to MASH (5), which can ultimately lead to cirrhosis and HCC (6). Liver fibrosis is the primary pathological feature of MASH. As MASH progresses, the severity of liver fibrosis gradually increases. Numerous studies aim to predict the degree of fibrosis in MASH to improve diagnosis and treatment strategies. Currently, there are very few clinically approved drugs available for the treatment of liver fibrosis, and the underlying mechanisms of liver fibrosis are still complex and not yet fully understood. Therefore, elucidating these molecular mechanisms and identifying potential new drug candidates is critical for halting the progression of MASH.

Cell death is a fundamental biological process that is integral to various life phenomena, including growth, development, aging, and disease (7). In 2018, the Cell Death Nomenclature Committee established guidelines addressing the morphological and biological aspects of cell death, classifying it into two primary types: accidental cell death (ACD) and RCD (8). ACD is an uncontrolled cell death process triggered by unexpected insult stimuli. These damaging stimuli exceed the regulatory capacity of the cell, ultimately leading to cell death. In contrast, RCD refers to the autonomous and orderly death of cells, which is controlled by genes to maintain the stability of the internal environment. The RCD that occurs under physiological conditions is called programmed cell death (PCD) (9). The liver serves as a crucial defense organ capable of responding to various pathogenic microorganisms and their products. Upon stimulation by damage-associated molecular patterns (DAMPs) and pathogen-associated molecular patterns (PAMPs), the liver activates a range of RCD modalities (10). Dead cells can mediate natural immunity or inflammatory responses, ultimately leading to the activation, proliferation, differentiation and secretion of extracellular matrix (ECM) by hepatic stellate cells (HSCs), leading to matrix deposition and fibrosis (11, 12). Different lethal subroutines during RCD influence the progression of MASH and the response to treatment. Therefore, understanding the role of cell death in fibrosis during the progression of MASH is essential for a comprehensive study of its fibrotic mechanisms and the identification of potential drug targets.

The immune system function in patients with MASH is compromised to varying degrees. During the progression of MASH, as fibrosis worsens, the characteristics of immune cells alter in accordance with the state of fibrosis (13, 14). Immune cells in the liver, including macrophages, T cells, B cells, and eosinophils, are implicated in various forms of RCD and play a role in the development of liver fibrosis. For instance, macrophages and eosinophils are involved in pyroptosis and necrosis, which in turn promote liver fibrosis (15, 16). However, the relationship between liver fibrosis and this phenomenon remains unclear and warrants further investigation. It follows that the immune microenvironment may serves as a crucial link between cell death and MASH.

In recent years, bioinformatics analysis and machine learning have gained recognition as effective strategies for the comprehensive and in-depth analysis of large datasets, such as transcriptome sequences (17). In this study, we selected two transcriptome datasets (GSE130970 and GSE49541) from the GEO database for our analysis. We used ten different types of machine learning to screen for key RCD-related DEGs. To further elucidate the changes in immune cells between early and late MASH samples, we conducted a cell classification analysis to investigate immune cell infiltration. Moreover, we validated the expression of the characteristic genes in MASH patients by the GSE89632 dataset and analyzed the correlation between the characteristic genes and the clinical features of MASH patients. Notably, we constructed mouse models of liver fibrosis induced by MCD diet or CCL4 to verify the expression changes of characteristic genes as fibrosis progresses. Overall, our findings reveal key genes related to RCD during the progression of liver fibrosis in MASH, which may serve as new targets for future clinical diagnosis and treatment of MASH patients, and provide a reference for liver fibrosis resulting from other causes.

## Materials and methods

### Data acquisition

According to Brunt staging, MASH can be classified into five stages: F0, F1, F2, F3, and F4. The GSE130970 dataset includes 23 patients in stage F0, 28 patients in stage F1, 9 patients in stage F2, 14 patients in stage F3, and 2 patients in stage F4. Additionally, the GSE49541 dataset comprises 40 patients in stages F0 and F1, as well as 32 patients in stages F2 through F4. In the subsequent analysis, we defined stages F0 and F1 as early MASH, while stages F2, F3, and F4 are categorized as late MASH. The GSE130970 and GSE49541 datasets were merged using the R packages limma and SVA (13, 18, 19). We first converted the RNA-seq data into log2(FPKM + 1) to approximate a normal distribution, while quantifying and normalizing the microarray data. For the initial mixed dataset, we first applied the combat function to eliminate batch effects, followed by the use of the preprocessCore software package in R to standardize the data. Ultimately, a mixed dataset comprising 91 early MASH samples and 57 late MASH samples was obtained. As a validation dataset, GSE89632 had 24 control samples, 19 MASH samples, and 21 simple steatosis (SS) samples, with the corresponding clinical information.

### Identifcation of DEGs

We utilized the R package “limma” to identify DEGs between early and late stages of MASH, applying thresholds of |logFC| ≥ 0.5 and adj.P. Val. < 0.05 for DEG identification. A list of key regulatory genes associated with 18 modes of RCD was compiled from a previous study (**Supplementary Table 1**) and intersected with the aforementioned differential genes, resulting in the identification of 55 key genes for subsequent analysis (20). The principal components analysis (PCA) plot, expression heatmap, and volcano plot of the DEGs were created using the “ggbiplot”, “pheatmap” and “ggplot2” packages via R software.

### Functional enrichment analysis

Functional enrichment analysis was conducted on the data to assess the potential functions of the identified targets. We performed enrichment analysis utilizing the KEGG and GO through the R package “clusterProfiler”. In these analyses, adjusted P values <0.05 were deemed statistically significant, and the top 20 results from each analysis were extracted for visualization.

### Machine learning models

We integrated ten diverse machine learning algorithms and evaluating 101 algorithmic combinations. Tese machine learning algorithms included Support Vector Machine (SVM), Least Absolute Shrinkage and Selection Operator (Lasso), Gradient Boosting Machine (GBM), Random Forest, Elastic Net, Stepwise Cox, Ridge, CoxBoost, Super Partial Correlation (SuperPC), and Partial Least Squares with Cox regression (plsRcox). We conducted 10-fold cross-validation on all suitable models. Additionally, we implemented several safeguard measures to prevent overfitting, which include regularization, early stopping, and sparsity. The GSE49541 dataset serves as the training set, while other datasets are utilized for validation. We calculated the area under the curve (AUC) as a criterion for optimal model selection. Correlations among variables were assessed using Pearson or Spearman correlation tests, with a significance threshold set at p < 0.05. Subsequently, we employed the receiver operating characteristic (ROC) curve and the AUC to evaluate the diagnostic efficacy of the characteristic genes.

### Immune infiltration analysis

To better identify immune cell signatures in the livers of MASH patients, we compared the differences in expression of immune cell types between the two groups using single-sample gene set enrichment analysis (ssGSEA). Pearson correlation analysis was used to reveal the correlation between immune cell distribution and DEGs expression, and the results were presented in the form of lollipop diagrams.

### Gene set enrichment analysis

GSEA is widely utilized to assess alterations in pathways and biological activities across samples within expression datasets. Based on the correlation analysis results between 11 individual genes and the entire gene set, we employed GSEA to conduct Reactome pathway enrichment analysis. The criterion for significance was set at a P value of < 0.05, and the top 20 pathways from each analysis were extracted for visualization.

### Drug gene interaction analysis

The Drug-Gene Interaction Database (DGIdb, www.dgidb.org) is a web resource that provides information on drug-gene interactions and druggable genes from publications, databases, and other web sources. We used the DGIdb database to predict drugs that may interact with DEGs.

### Consensus clustering analysis

Hierarchical clustering analysis was conducted on 150 MASH samples utilizing the “Consensus Clustering” R package, with the expression profiles of 11 characteristic DEGs serving as input data. A PCA plot was generated to illustrate the differences between subgroups A and B, employing the ggplot2 package. Additionally, box plots and heat maps were utilized to depict the expression differences of the 11 characteristic genes in type 2. We downloaded the hallmark, KEGG, and reactome pathways from the Msigdb database and subsequently employed the R package GSVA to perform pathway scoring to compare the pathway differences between the two subtypes. Heatmaps comparing the two groups were drawn using the R package pheatmap.

### Animals and treatment

Male C57BL/6 mice (8 weeks old) were purchased from Beijing HuaFuKang Biotechnology Co., Ltd. and maintained under specifc pathogen-free conditions in the Animal Care Facility of East China Normal University. All the mice were used in compliance with the guidelines of Institutional Animal Care and Use Committee of East China Normal University. The experiment was divided into 3 groups (CON, MCD 4w, MCD 8w), with 6 mice in each group. To induce MASH, mice were fed with MCD diet for 4 or 8weeks. Liver fibrosis in mice was induced by intraperitoneal injection of 2 ml/kg body weight of 20% CCL4 dissolved in mineral oil, twice a week for up to 4 or 8weeks. This experiment is divided into 3 groups (CON, CCL4 4w, CCL4 8w), with 6 mice in each group.

### Cell culture and treatment

The human hepatic stellate cell line LX2 was derived from the Cell Bank of the Chinese Academy of Sciences (Shanghai, China). Cells were seeded in culture plates and dishes at appropriate densities and cultured in DMEM supplemented with 10% fetal bovine serum (FBS). Once the confluence reached 80-90%, LX2 cells were treated with 10 ng/mL TGF-β1 for 24 hours. Recombinant Human TGF-β1 (Cat# 100–21) was purchased from Peprotech. To further investigate the influence of EPHA3 on the activation of LX2 cells, we employed siRNA interference to knock down the expression of EPHA3 (**Supplementary Table 2**).

### Histopathological staining

Livers were excised and immediately fixed with 10% bufered formalin. Samples were embedded in parafn and cut into 5 μm sections. Liver sections were deparafnized, rehydrated, and routinely stained with hematoxylin and eosin (HE) or Sirius Red. Frozen liver sections were cut into 10 μm sections. Tissues were fixed in acetone for 10 min and then washed in PBS for 3 min. After fixation, tissue was washed with PBS and subjected to Oil red staining.

### Real-time PCR

Total RNA was extracted with Trizol reagent and then reverse transcribed using the Tian Gen Biotech (Beijing, China). Amplifcation was performed using the Power SYBR^®^ Green PCR Master Mix (Applied Biosystems) according to the manufacturer’s instruction. The relative expression level of each transcript was normalized to murine GAPDH by using the 2 (ΔΔCt) method. The primers were listed in **Supplementary Table 2**.

### Western blot

Proteins were extracted with radioimmunoprecipitation (RIPA) lysis bufer with protease inhibitor cocktail (#04693132001, Roche Applied Science, Mannheim, Germany). Lysates containing equal amounts of protein were separated by 10% SDS-PAGE and transferred to a polyvinylidene difluoride membrane (Millipore). The membrane was incubated with 5% skim milk for 1 h and then incubated with primary antibodies at 4°C overnight. Primary antibodies against the following proteins were used: β-actin (#66009, Proteintech), α-SMA (ab7817, Abcam), EPHA3 (PA1391, Abmart), Fibronecting (#15613 Proteintech), p-Smad3 (#9520, CST), Smad3 (#9520, CST).

### Statistical analysis

All statistical tests were conducted using R software version 4.2.2. The Wilcoxon test or Student’s t-test was employed to analyze differences between the two groups. Correlations between variables were assessed using either Pearson or Spearman correlation tests. All p-values are two-sided, with a significance level set at *p* < 0.05.

## Results

### Identification of differentially expressed genes and enrichment analysis

**Figure 1:**
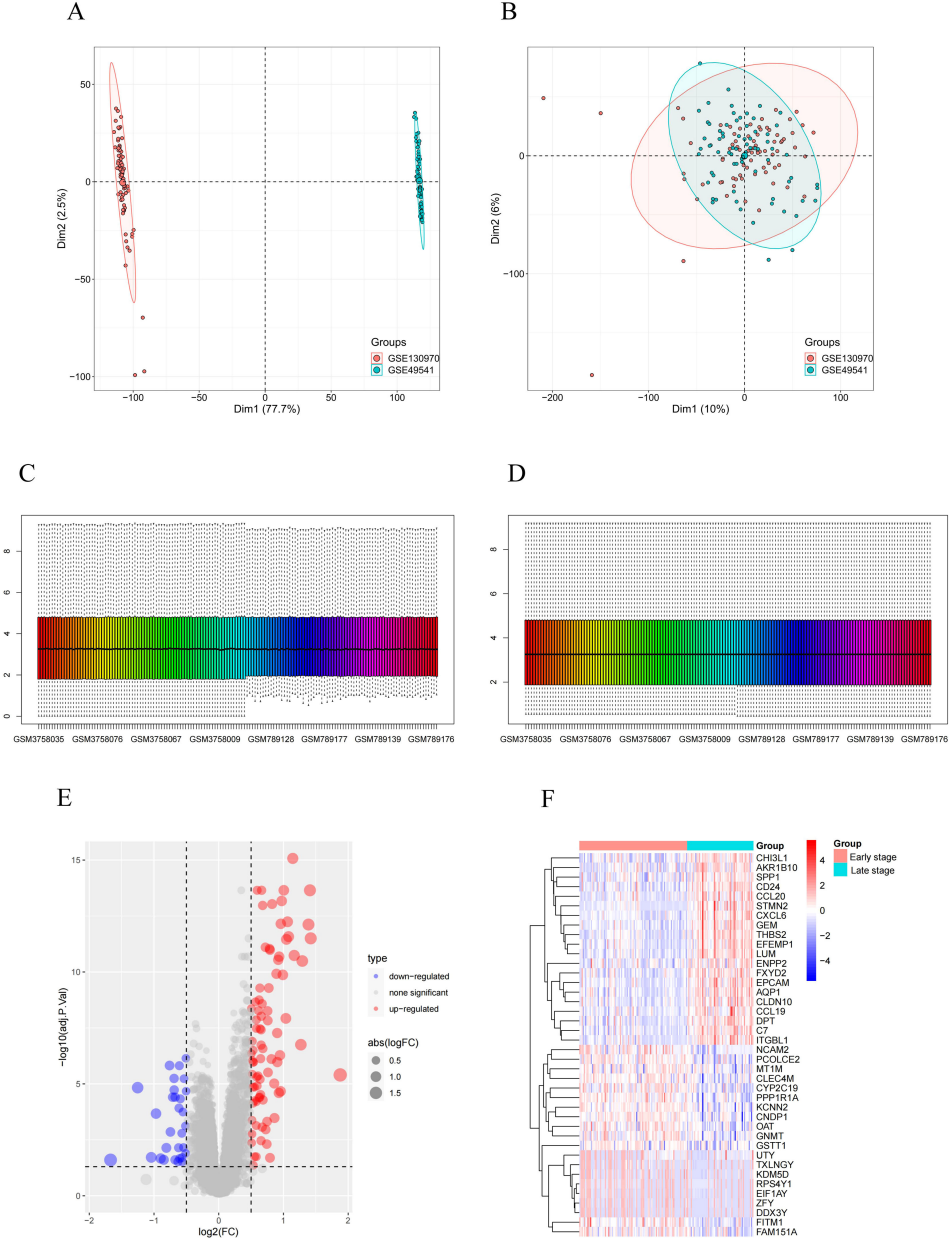
Identification of DEGs. **(A)** PCA cluster plot of GSE130970 and GSE49541 before batch effect removal and correction. **(B)** PCA cluster plot showed that batch effect has been removed. **(C)** Box plots of the original data before normalization. **(D)** Box plots of the original data after normalization. **(E)** Volcano plot of DEGs between early-stage and late-stage MASH patients. **(F)** Heatmap for the DEGs between early-stage and late-stage MASH patients. Red: Up-regulation; Green: Down-regulation.

In this study, two high-throughput sequencing datasets, GSE130970 and GSE49541, were normalized and merged into one dataset. Subsequently, the merged dataset was subjected to batch effect elimination before data analysis. The PCA plot shows that before the removal of the batch effect, the sample distribution of each dataset varied significantly, indicating the presence of a batch effect (**Figure 1A**). After applying SVA and Limma correction, samples from different batches overlapped, indicating a substantial reduction in batch effects across two gene sets (**Figure 1B**). Next, we normalized the merged dataset to eliminate the adverse effects of singular sample data (**Figures 1C, D**). The fibrosis stages of MASH are classified as follows: F0 indicates no fibrosis, F1 represents perisinusoidal fibrosis, F2 denotes perisinusoidal fibrosis combined with portal area fibrosis, F3 refers to bridging fibrosis, and F4 signifies cirrhosis. In our classification, F0 and F1 are categorized as early MASH, while F2 through F4 are classified as late MASH. The final training dataset consisted of an early MASH group (including 85 patients) and an advanced MASH group (including 91 patients). Using the limma differential gene analysis package in R, we identified 115 DEGs in the merged dataset, comprising 85 up-regulated genes and 30 down-regulated genes. **Figures 1E, F** illustrate volcano plots and heatmaps of the DEGs between the early and late stages of MASH in the merged dataset.

**Figure 2:**
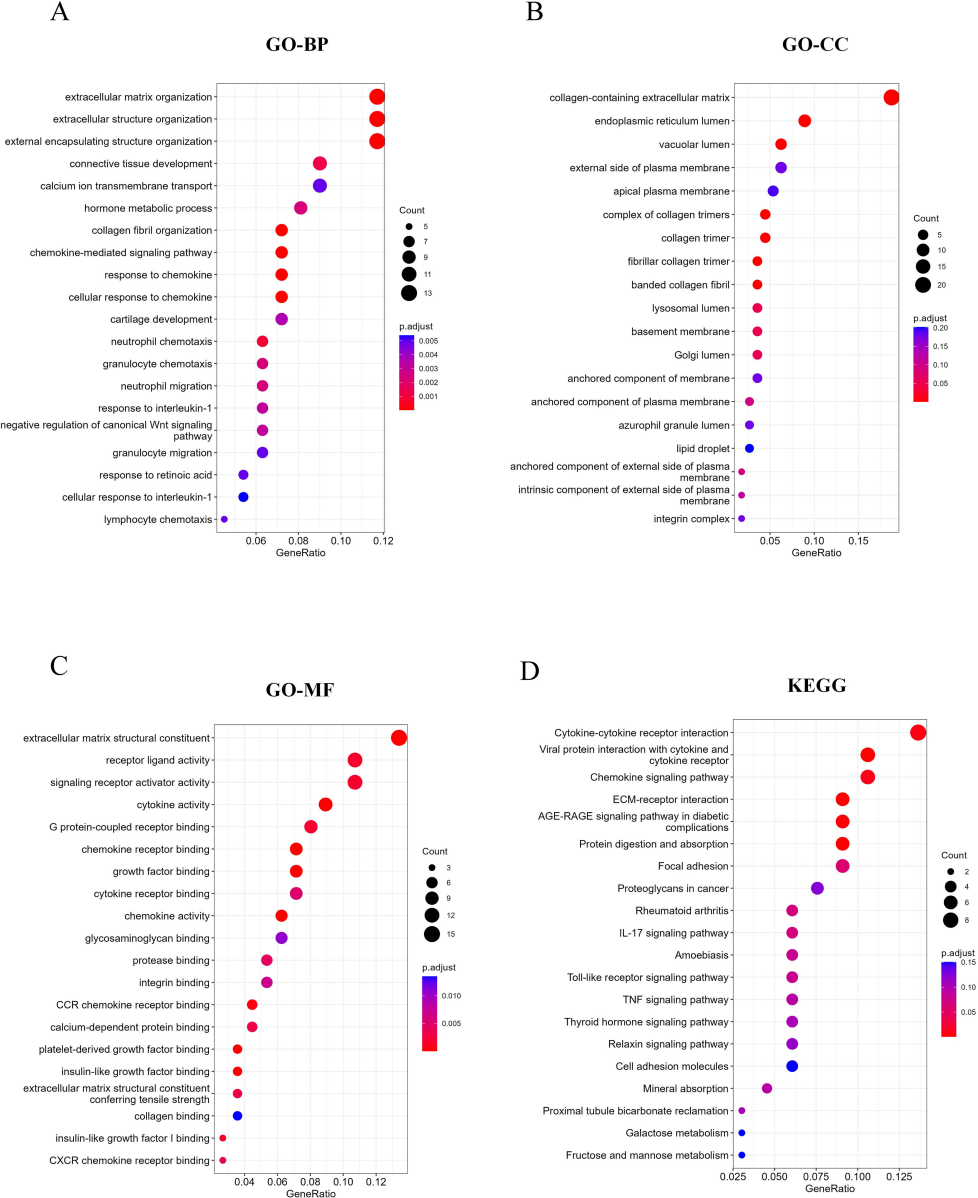
Function and pathway enrichment analysis of DEGs. **(A-C)** Top 20 enriched biological functions of DEGs determined by GO. **(D)** Top 20 enriched KEGG pathways of DEGs determined by KEGG pathway enrichment analysis.

To investigate the role of these DEGs in MASH, we conducted GO and KEGG pathway enrichment analyses. As illustrated in **Figures 2A-C**, the GO results indicate that DEGs are predominantly enriched in ECM-related pathways, including extracellular matrix organization and structural organization. Furthermore, immune-related pathways, such as chemokine-mediated signaling pathways, cellular responses to chemokine stimulation, and granulocyte migration, were also found to be enriched. The KEGG analysis revealed additional enrichment in cytokine-cytokine receptor interactions, ECM-receptor pathways, chemokine signaling pathways, and TNF signaling pathways (**Figure 2D**). Collectively, these findings suggest that both ECM-related and immune-related pathways play significant roles in the occurrence and progression of MASH.

### Identification of RCD-related DEGs and enrichment analysis

**Figure 3:**
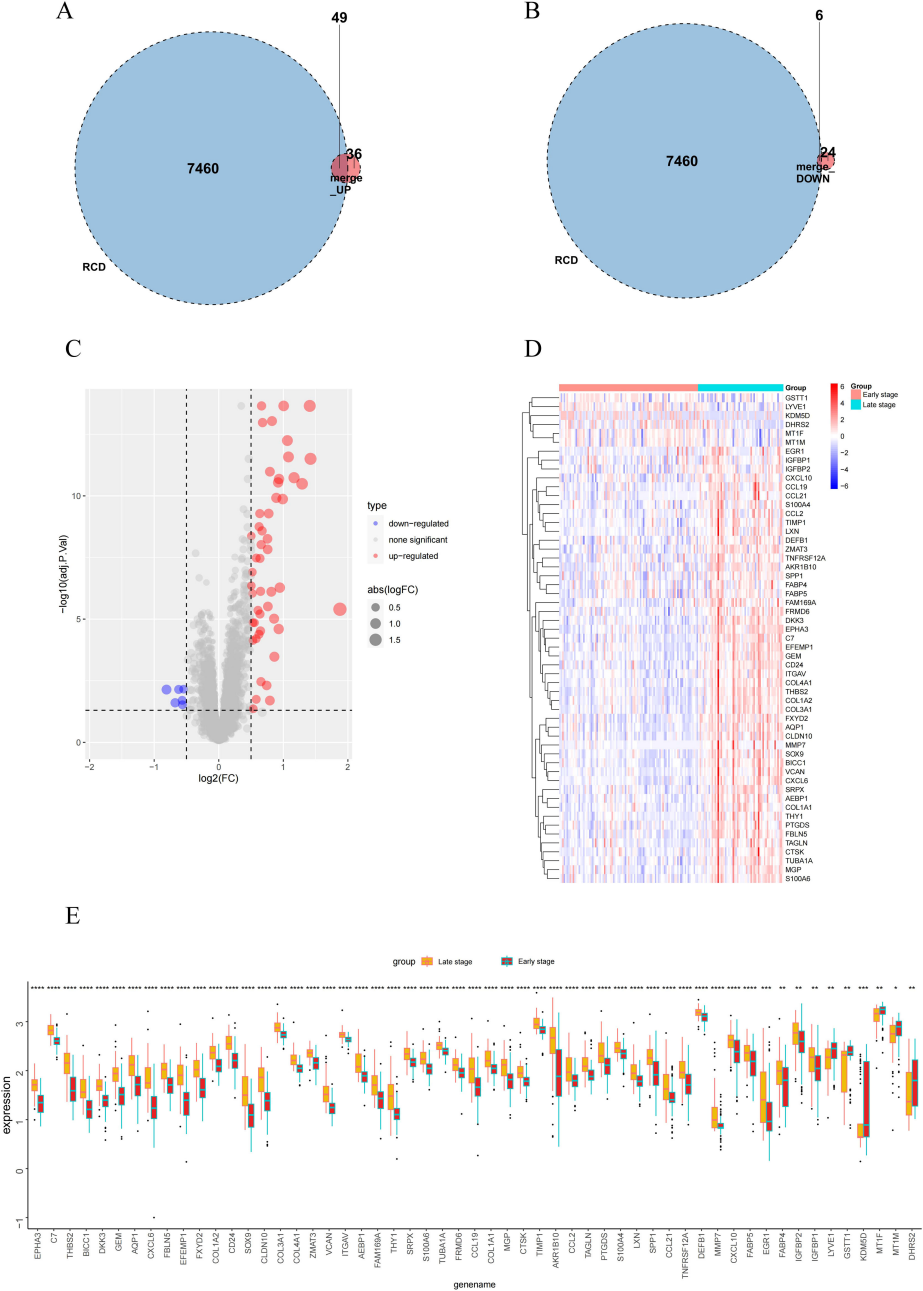
RCD-related DEGs expression and enrichment analysis in early-stage and late-stage groups. **(A, B)** Venn diagram of the intersection of RCD-related genes and DEGs. **(C-E)** Volcano plot, heatmap plot and box plot of the RCD-related DEGs. Red: Up-regulation; Green: Down-regulation. (*, P < 0.05; **, P < 0.01; ***, P < 0.001; ****, P < 0.0001).

**Figure 4:**
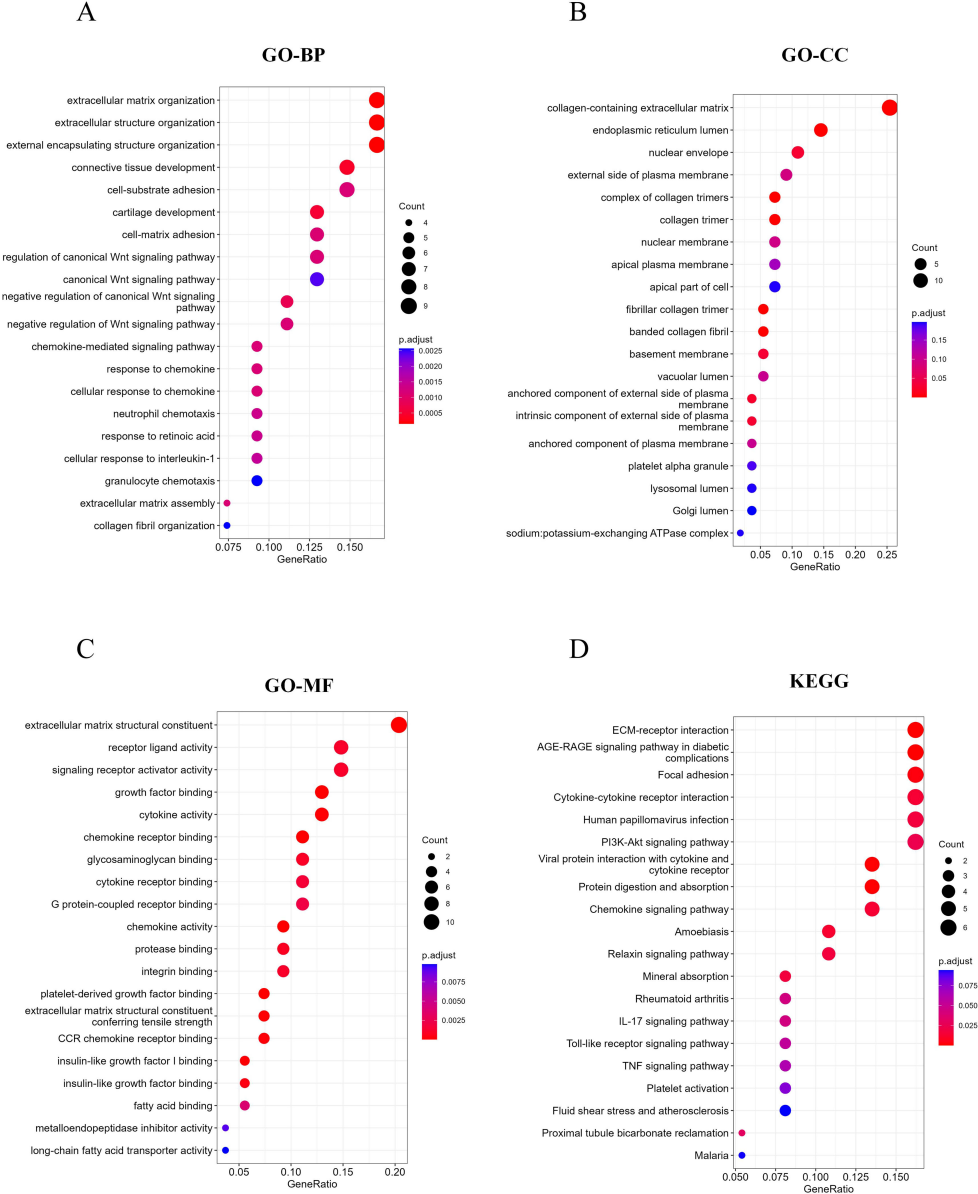
RCD-related DEGs enrichment analysis. **(A-C)** Top 20 enriched biological functions of DEGs determined by GO. **(D)** Top 20 enriched KEGG pathways of DEGs determined by KEGG pathway enrichment analysis.

Studies have shown that the development of MASH is accompanied by a variety of RCD. RCD regulates the infiltration of immune cells and plays a key role in the fibrogenesis and inflammatory damage of MASH. Important regulatory genes containing 18 RCD modes have been collected in the literature (20). Subsequently, the 115 differential genes identified were intersected with 7,460 RCD-related genes (**Supplementary Table 1**), resulting in 55 overlapping differential genes, which included 49 up-regulated genes and 6 down-regulated genes (**Figures 3A-E**). Following this, we performed GO and KEGG enrichment analyses on the samples based on RCD-related DEGs to investigate the biological characteristics of these characterized differential genes (**Figures 4A-D**). The results showed that RCD-related DEGs were relatively enriched in inflammatory processes, extracellular matrix organization, and immune-related pathways.

### Identifying characteristic RCD-related DEGs by machine learning

**Figure 5:**
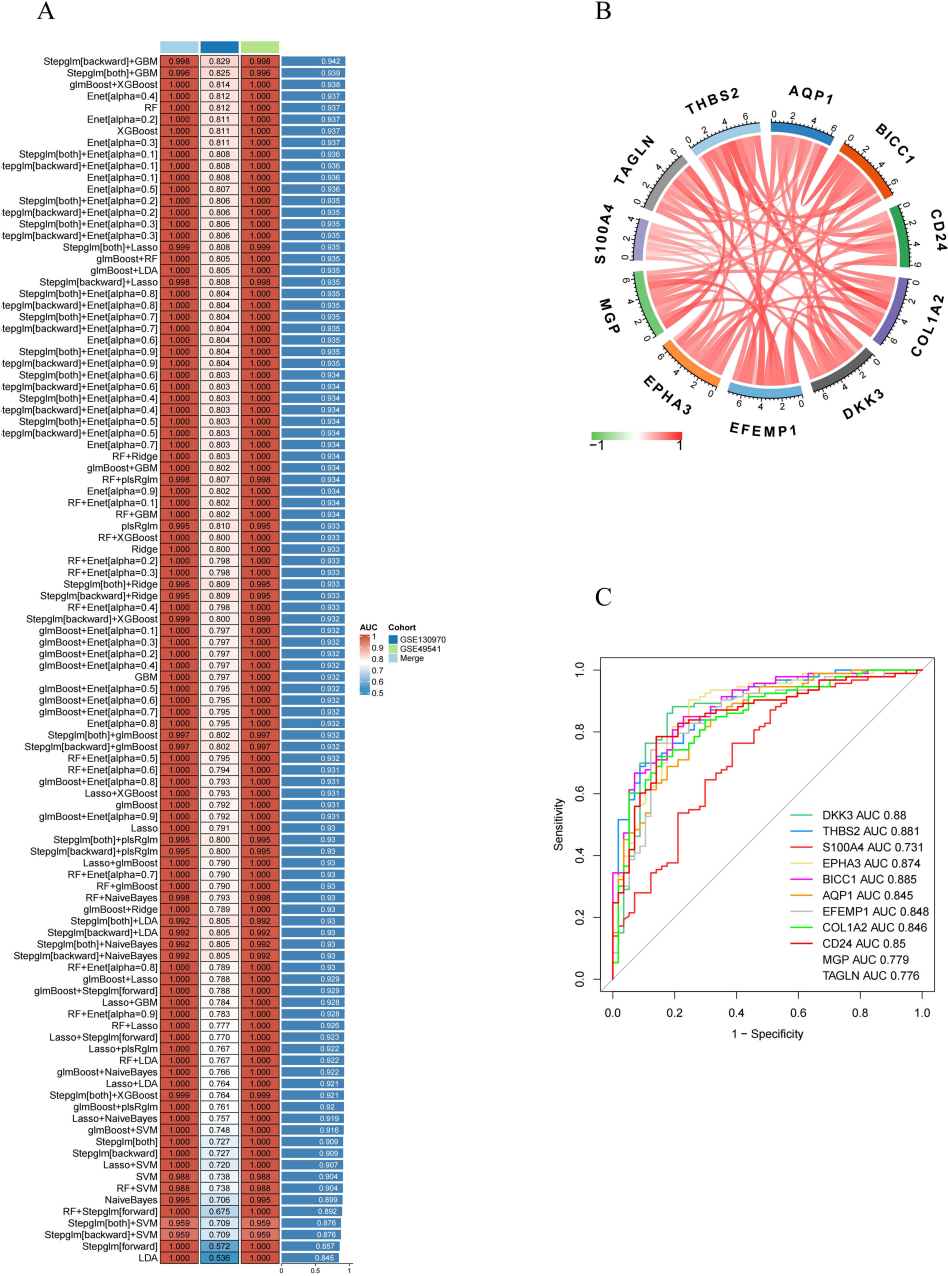
Identification of feature RCD-related DEGs via machine learning. **(A)** A total of 101 kinds of prediction models via a tenfold cross-validation framework and further calculated the C index of each model across all validation datasets. **(B)** Chord diagram displaying the relationship between the feature RCD-related DEGs. **(C)** ROC curve of the feature RCD-related DEGs in late-stage MASH diagnosis.

Based on 55 RCD-related DEGs with predictive significance, we employed 10 machine learning algorithms to construct a diagnostic model. Using the GSE49541 dataset as the training set, we developed 101 prediction models (**Figure 5A**) and assessed the performance of each model on additional independent validation sets. Subsequently, we calculated AUC for all training and validation sets, selecting the genes associated with the top three machine learning algorithms that exhibited the highest AUC. This process yielded a total of 11 characteristic genes.

Subsequently, we analyzed the correlation among 11 characteristic DEGs to investigate the potential role of RCD in the progression of MASH. The results indicated a strong correlation among these 11 genes (**Figure 5B**). Furthermore, the expression levels of these characteristic genes, as depicted in the figure, were significantly higher in late-stage MASH compared to early-stage MASH (**Figure 3C**), suggesting their potential involvement in the progression of the disease.We also evaluated the diagnostic performance of each signature gene in predicting the progression of MASH within the combined GSE130970 and GSE49541 cohorts. Among these genes, BICC1 exhibited the highest AUC value, reaching 0.885. The AUC values for THBS2, DKK3, EPHA3, CD24, EFEMP1, COL1A2, AQP1, MGP, TAGLN and S100A4 were 0.881, 0.88, 0.874, 0.85, 0.848, 0.846, 0.845, 0.779, 0.776 and 0.731, respectively (**Figure 5C**). These results indicate that these signature genes can effectively estimate the progression of MASH.

### Evaluation of immune cell infiltration in MASH

**Figure 6:**
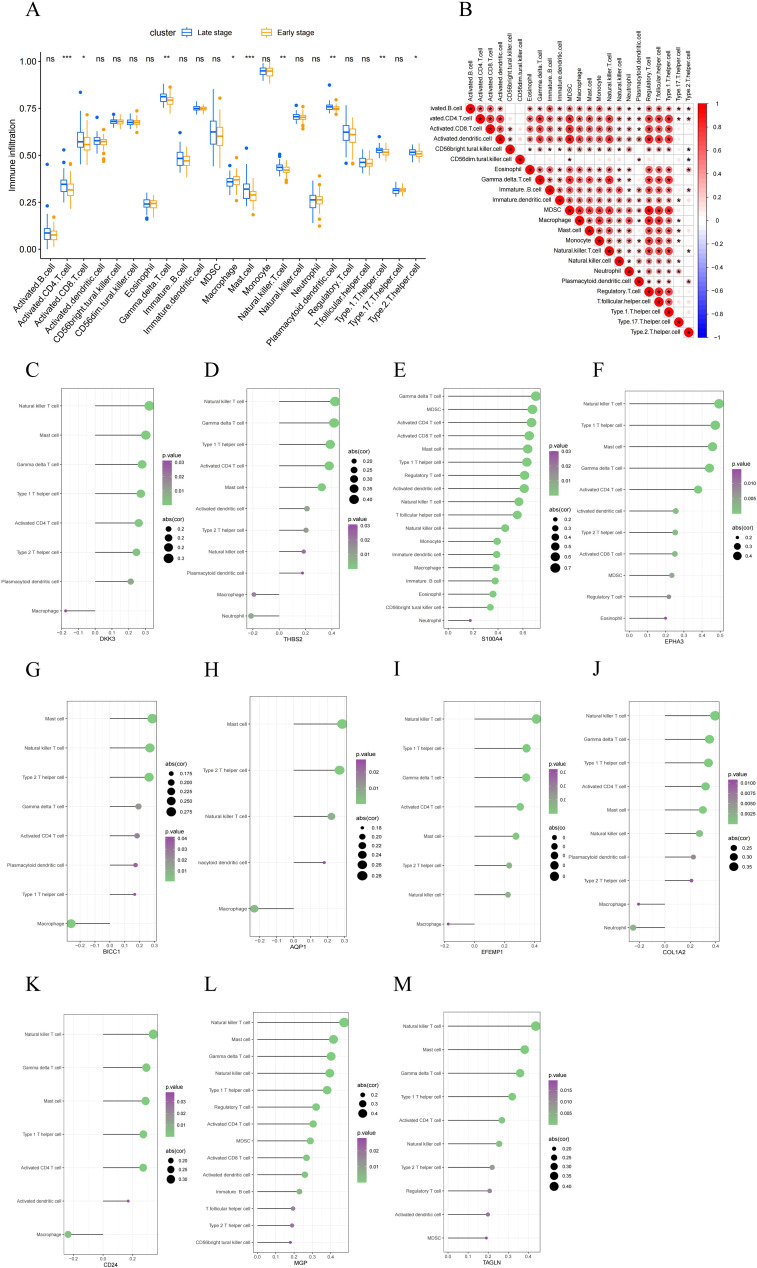
Evaluation of immune cell infiltration. **(A)** A box plot comparing the infiltration rates of immune cells between early-stage and late-stage MASH groups. **(B)** Correlation between 22 distinct populations of immune cells. The red color represents a positive correlation. **(C–M)** Correlation analysis between feature genes and immune cells. A larger circle shows a stronger correlation. A higher density of green color shows a more robust association. (*, P < 0.05; **, P < 0.01; ***, P < 0.001; ns, no statistical signifcance.).

We evaluated the immune microenvironment of MASH patients by analyzing immune cell infiltration. Compared with early-stage MASH, most innate and adaptive immune cells presented higher infiltration levels in advanced-stage MASH (**Figure 6A**). Moreover, there were remarkable interactions between immune cell populations across MASH (**Figure 6B**). These findings suggested that advanced MASH is associated with a more robust immune response. Subsequently, we conducted a further investigation into the correlation between 11 key RCD-related DEGs and immune cell infiltration. The results of the correlation analysis revealed a significant interaction between the signature genes and immune cell infiltration (**Figures 6C-M**). Notably, S100A4, EPHA3 and MGP were found to be closely associated with the infiltration of the greatest variety of immune cells. These results indicate that the key RCD-related DEGs may play a regulatory role in immune characteristics during the progression of MASH.

### Single-gene enrichment analysis of characteristic RCD-related DEGs

**Figure 7:**
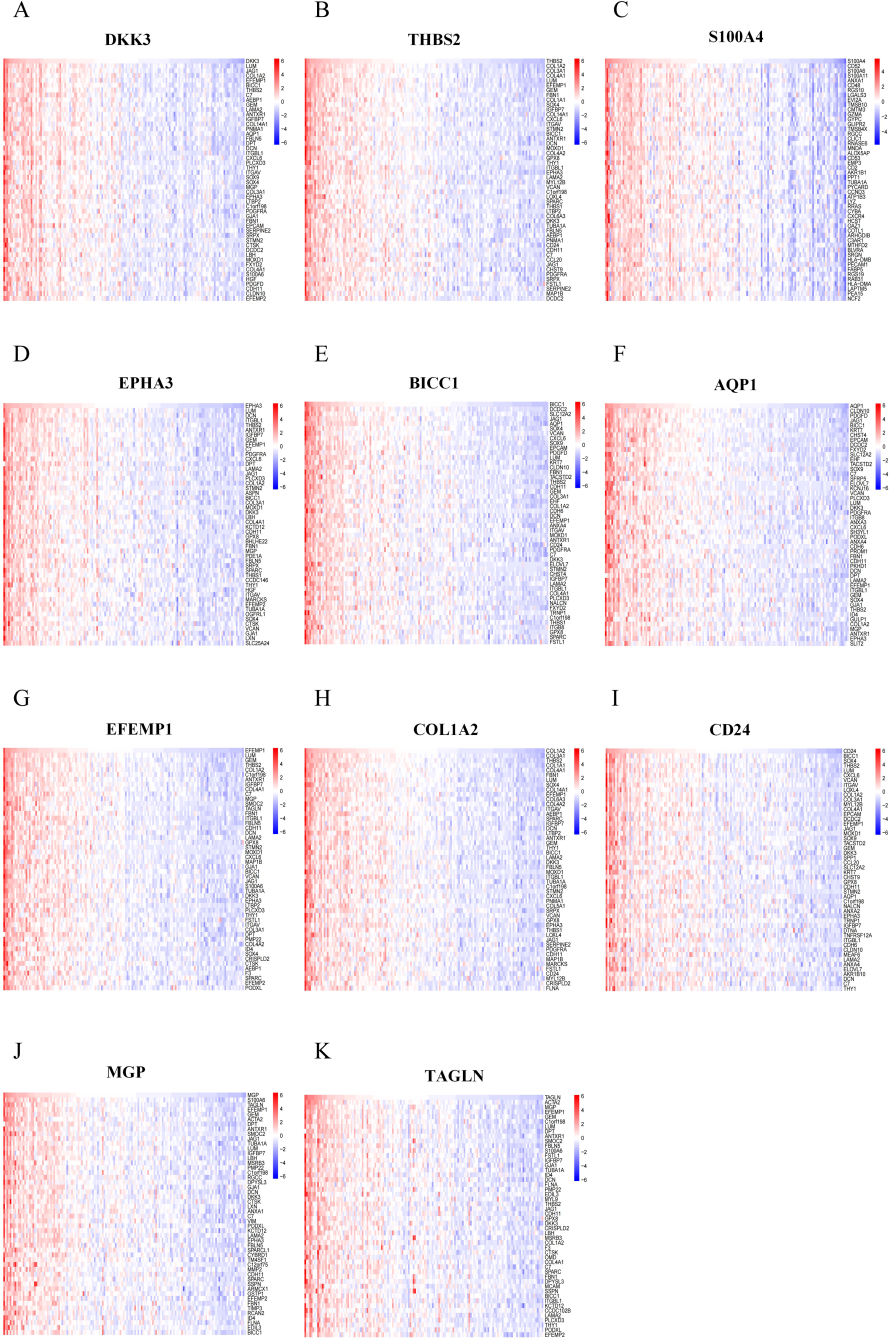
Correlation analysis of feature RCD-related DEGs with all genes. **(A-K)** Correlation analysis of 11 RCD-related DEGs with all genes was performed using heatmaps to show the expression of positively correlated top50 genes, respectively.

**Figure 8:**
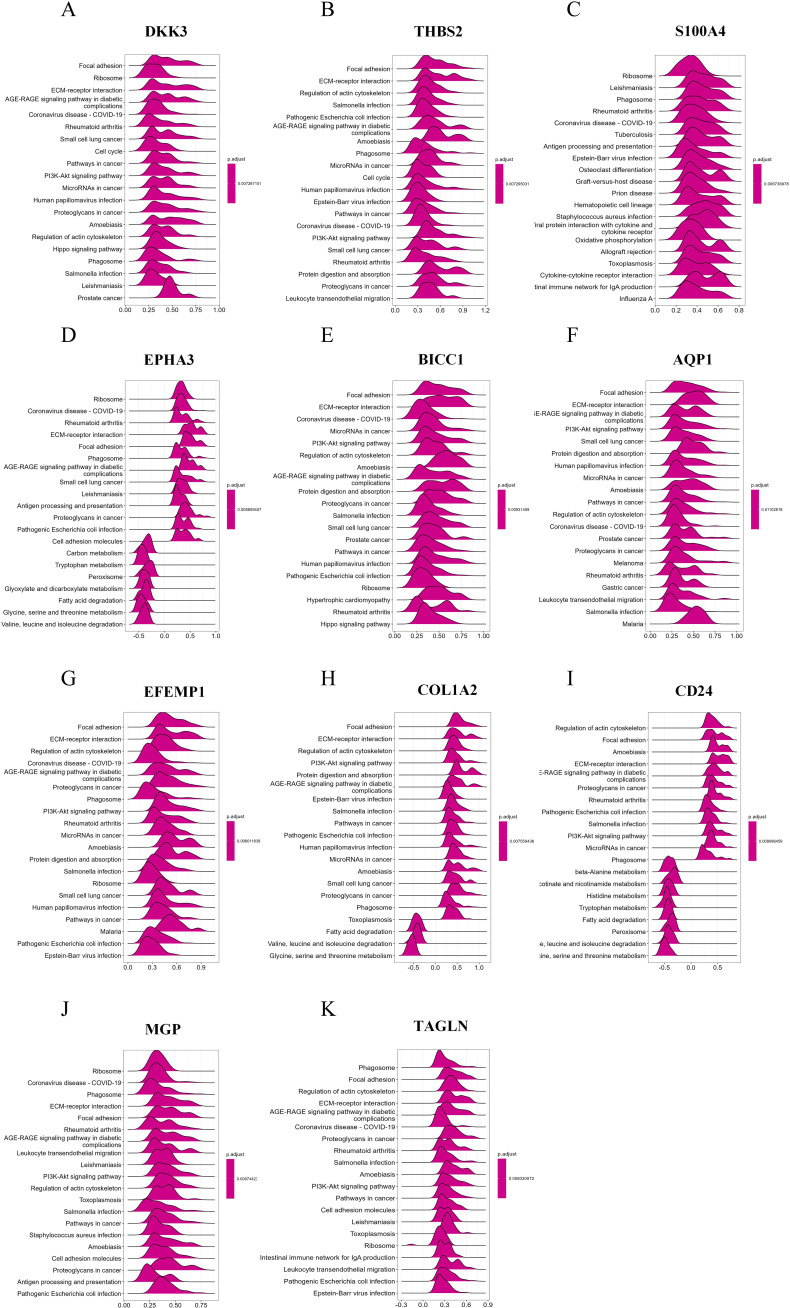
Single-gene GSEA of feature RCD-related DRGs. **(A-K)** Top 20 enriched reactome pathways of the key RCD-related DRGs determined by GSEA.

RCD can be triggered by the stimulation of DAMPs and PAMPs. The presence of some dead cells elicits inflammatory responses, facilitates innate immunity, and prompts myofibroblasts to secrete ECM, resulting in matrix deposition and fibrosis. To investigate the biological significance of core genes, we analyzed the correlation between 11 key genes and the entire gene set, employing a heat map to illustrate the expression of the top 50 positively correlated genes (**Figures 7A-K**). As shown in the heatmap, the majority of positively correlated genes are associated with inflammatory chemokines (such as CXCL6 and CCL2) and fibrotic factors (including COL1A2, COL3A1, and COL4A1). Building on the results of the correlation analysis, we employed the GSEA algorithm to further investigate the functions associated with the core genes. The **Figures 8A-K** present the top 20 results from the reactome pathway derived from 11 single-gene GSEA analyses. GSEA results indicate that the expression levels of AQP1, BICC1, DKK3, CD24, COL1A2, EFEMP1, EPHA3, TAGLN, THBS2, and MGP are primarily positively associated with ECM organization and ECM proteoglycans. Additionally, the expression of AQP1, COL1A2, THBS2, and CD24 shows a positive correlation with collagen formation. The expression levels of AQP1, COL1A2, BICC1, DKK3, EPHA3, TAGLN, MGP, THBS2, CD24, and EFEMP1 are positively correlated with the formation of elastic fibers. In contrast, S100A4 is primarily associated with neutrophil degranulation and the innate immune system. These indicate that these RCD-related genes play a significant role in the development of MASH fibrosis.

### MASH unsupervised clustering identification and analysis

**Figure 9:**
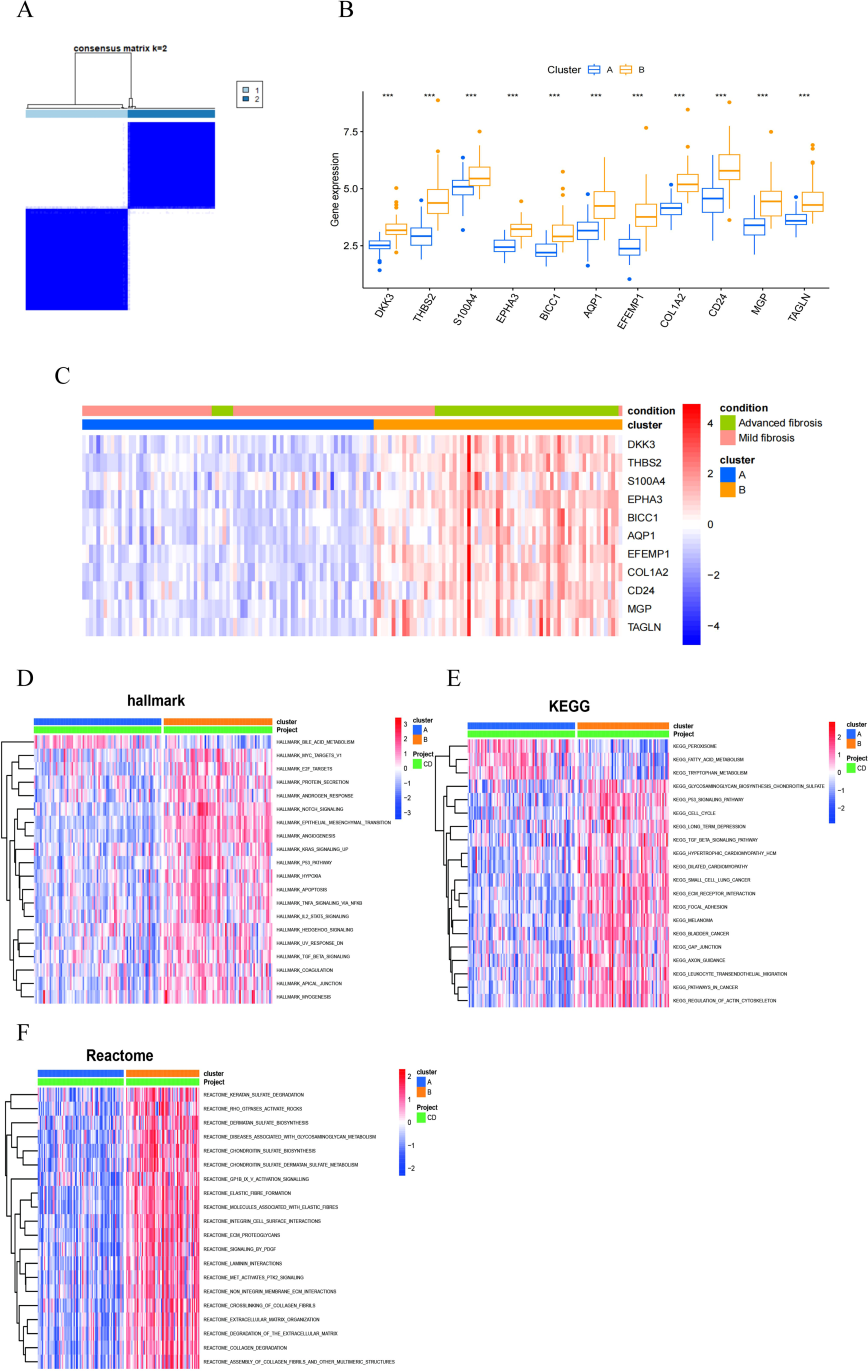
MASH classifcation based on the RCD-related DEGs. **(A)** The consensus clustering with k=2. **(B)** A box plot showed the expression of 11 RCD-related DEGs between two diferent subgroups. **(C)** Heatmap for the the expression of 11 RCD-related DEGs between two diferent subgroups. **(D-F)** Pathway activity between two diferent subgroups based on GSVA. (***, P < 0.001).

To explore the predictive potential of RCD-related genes in MASH patients, we performed unsupervised cluster analysis utilizing the R package “ConsensusClusterPlus” by varying the clustering variable k from 2 to 10, it is found that when k = 2, the intra-group correlation is the highest and the inter-group correlation remained low (**Figure 9A**). This finding suggested that the 176 MASH patients can be effectively categorized into two distinct clusters based on the aforementioned 11 DEGs. The expression of all characteristic genes was higher in cluster 2 than in cluster 1 (**Figure 9B**). The heat map results also demonstrate the expression levels of these 11 genes in both cluster 1 and cluster 2, revealing significant differences between the two groups. Notably, there was a marked difference in the degree of fibrosis between the subgroups, with the group exhibiting high expression of the signature gene showing a greater extent of fibrosis (**Figure 9C**).

In order to explore the differences between different pattern clustering in biological processes mediated by characteristic genes, we downloaded the hallmark, KEGG, and reactome pathways from the Msigdb database. Subsequently, we performed pathway scoring using the R package GSVA and identified significant differences in pathways between the two clusters. The Hallmark pathway analysis revealed that cluster 2 is predominantly enriched in the Notch signaling pathway, TGFβ signaling pathway, Hedgehog signaling pathway, TNFα-NF-κB signaling pathway, epithelial-mesenchymal transition (EMT) signaling pathway, apoptosis, and hypoxia (**Figure 9D**). The KEGG pathway analysis reveals that cluster 1 is primarily enriched in peroxisomes, fatty acid metabolism, and tryptophan metabolism, while cluster 2 also shows significant enrichment in the p53 signaling pathway, TGFβ signaling pathway, ECM receptors, and leukocyte migration (**Figure 9E**). Reactome pathway analysis indicated that cluster 2 was predominantly enriched in extracellular matrix tissue, ECM proteoglycans, the PDGF signaling pathway, elastic fibers, and collagen fibers (**Figure 9F**). Consequently, utilizing these 11 RCD-related DEGs, MASH patients can be categorized into two distinct groups, exhibiting significant differences in fibrosis-related characteristics.

### The correlation between RCD-related DEGs ang clinical features of MASH patients

**Figure 10:**
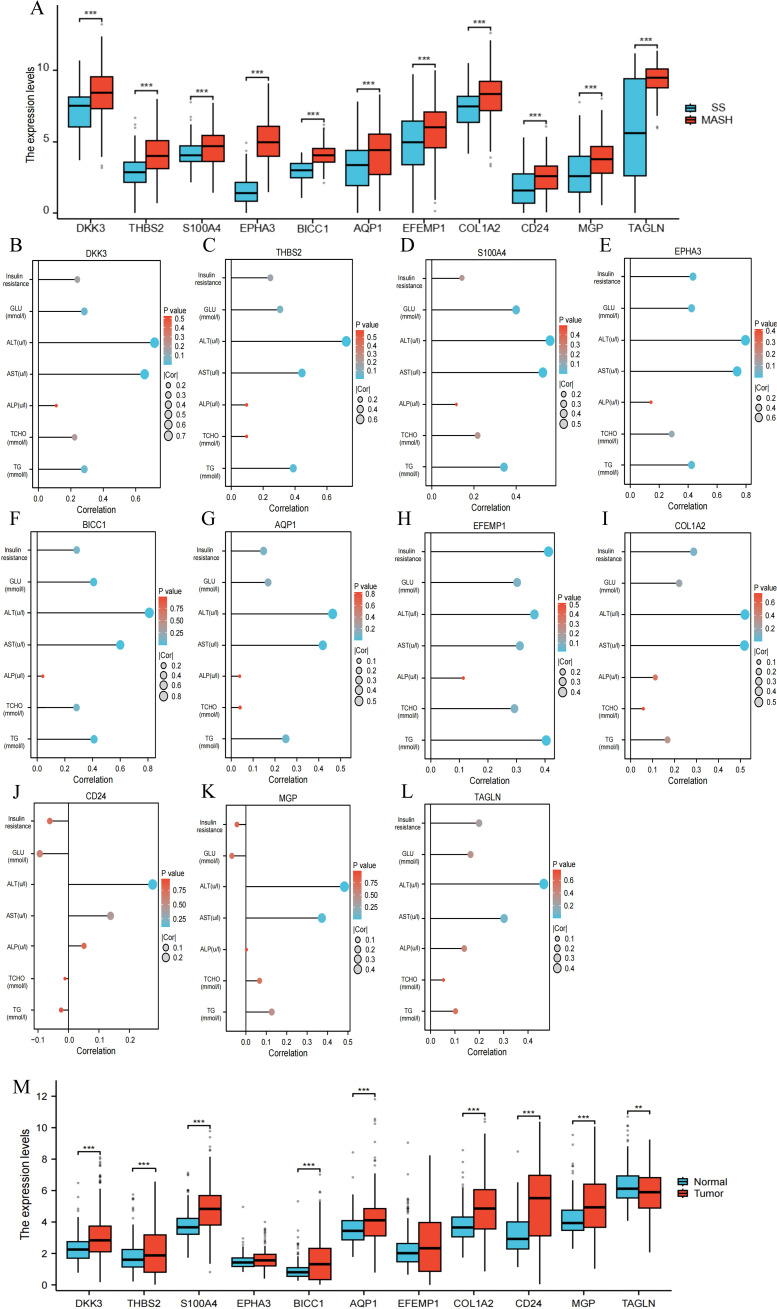
The correlation between RCD-related DEGs and clinical features of MASH. **(A)** Expression of the RCD-related DEGs in the GSE89632 dataset. **(B-L)** Spearman correlation analysis of the RCD-related DEGs with MASH clinical variables. Larger circles indicate stronger correlations. Higher density of blue color indicates stronger correlation. **(M)** Expression of the RCD-related DEGs in HCC. (**, P < 0.01; ***, P < 0.001.).

We validated 11 RCD-related DEGs using the GSE89632 dataset and found that the characteristic genes were significantly upregulated in patients with MASH compared to those with SS (**Figure 10A**). Additionally, we analyzed the relationship between changes in gene expression and clinical variables, discovering that AST and ALT levels were positively correlated with the expression of the signature genes (**Figures 10B-L**). It is well-known that the degree of liver dysfunction is closely associated with the severity of MASH. MASH is a more severe form of MAFLD. Among patients with MASH-induced cirrhosis, the estimated annual incidence of HCC ranges from 0.5% to 2.6% (4, 21). To further investigate the relationship between the signature genes and HCC, we analyzed the expression of key genes in HCC tissues and adjacent non-cancerous tissues. Our findings indicated that the majority of the DEGs are significantly upregulated in HCC tissues (**Figure 10M**). These results illustrate that characteristic RCD-related DEGs identified in our study not only have the potential to predict liver function impairment in patients with MASH but may also serve as biomarkers for HCC.

### Prediction of drug-gene interactions

Next, we investigate the potential of these signature genes as drug targets for the treatment of MASH. Drug-gene interaction data from the DGIdb database revealed 64 candidate therapeutic drugs or compounds for MASH. Here, the top 30 drugs/compounds ranked according to their “interactome score” from the DGIdb database were presented (**Figure 8C**). Among these, 12 drugs target AQP1 with high prediction scores; 7 drugs target EPHA3; 5 drugs target COL1A2; and 2 drugs each target THBS2, S100A4, and TAGLN, respectively. Notably, no potential drugs have been identified for treating BICC1, DKK3, CD24, EFEMP1, and MGP (**Table 1**).

**Table 1:** Drug–gene interaction prediction of RCD-related DEGs.

gene2	drug
EPHA3	IFABOTUZUMAB
EPHA3	KB-004
THBS2	CORTICOTROPIN
COL1A2	ST034307
EPHA3	COMPOUND 7 [WO2012007375]
EPHA3	COMPOUND 1 [WO2012007375]
EPHA3	IRAK-1/4 INHIBITOR
AQP1	CARBONIC ANHYDRASE INHIBITOR
TAGLN	RECOMBINANT TRANSFORMING GROWTH FACTOR-BETA 1
COL1A2	RECOMBINANT TRANSFORMING GROWTH FACTOR-BETA-2
S100A4	CHEMBL: CHEMBL585502
EPHA3	TAKINIB
AQP1	J-2156
AQP1	L-803,087
AQP1	H-C[DCYS-PHE-LAGL(N&BETA;ME,BENZOYL)-DTRP-LYS-THR-PHE-CYS]-OH
AQP1	BIM 23295
AQP1	&BETA;3-TETRAPEPTIDE
AQP1	NNC269100
AQP1	H-C[CYS-PHE-LAGL(N&BETA;ME,BENZOYL)-DTRP-LYS-THR-PHE-CYS]-OH
AQP1	H-C[CYS-PHE-LAGL(N&BETA;ME,BENZOYL)-TRP-LYS-THR-PHE-CYS]-OH
THBS2	BEVACIZUMAB-AWWB
TAGLN	AZACITIDINE
AQP1	[125I]TYR11-SRIF-14
AQP1	ANALOG 32 [PMID:18543899]
AQP1	REMLARSEN
COL1A2	COLLAGENASE CLOSTRIDIUM HISTOLYTICUM-AAES
EPHA3	BELIZATINIB
COL1A2	RECOMBINANT TRANSFORMING GROWTH FACTOR
S100A4	DAUNORUBICIN HYDROCHLORIDE
COL1A2	TNF-ALPHA

### The expression level of RCD-related DEGs in mice with liver fibrosis

**Figure 11:**
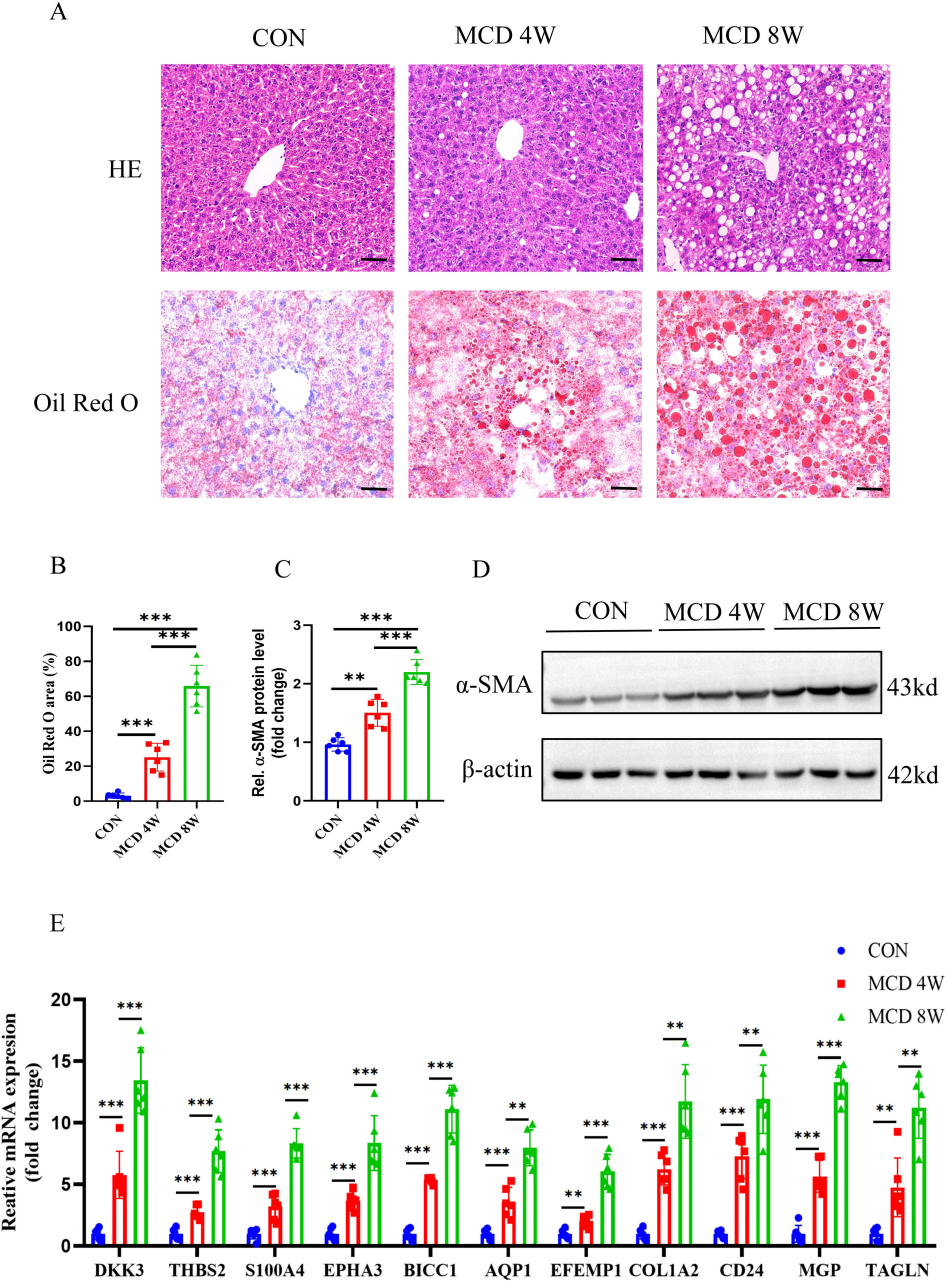
Animal experiment. **(A)** H&E and Oil Red O staining of liver sections from mice fed with control or MCD-diet (4 or 8weeks). Scale bar: 50 mm. **(B)** Semiquantitative analysis of Oil Red O area. **(C)** Semiquantitative measurement for the protein levels in **(D)** Western blot assay showed the protein expression of α-SMA in the liver from mice fed on the control or MCD-diet (4 or 8 weeks). **(E)** Determination of the key RCD-related DEGs expression in the liver of the control group or MCD-diet mice by RT-PCR (n=6 per group). (**, P < 0.01; ***, P < 0.001).

**Figure 12:**
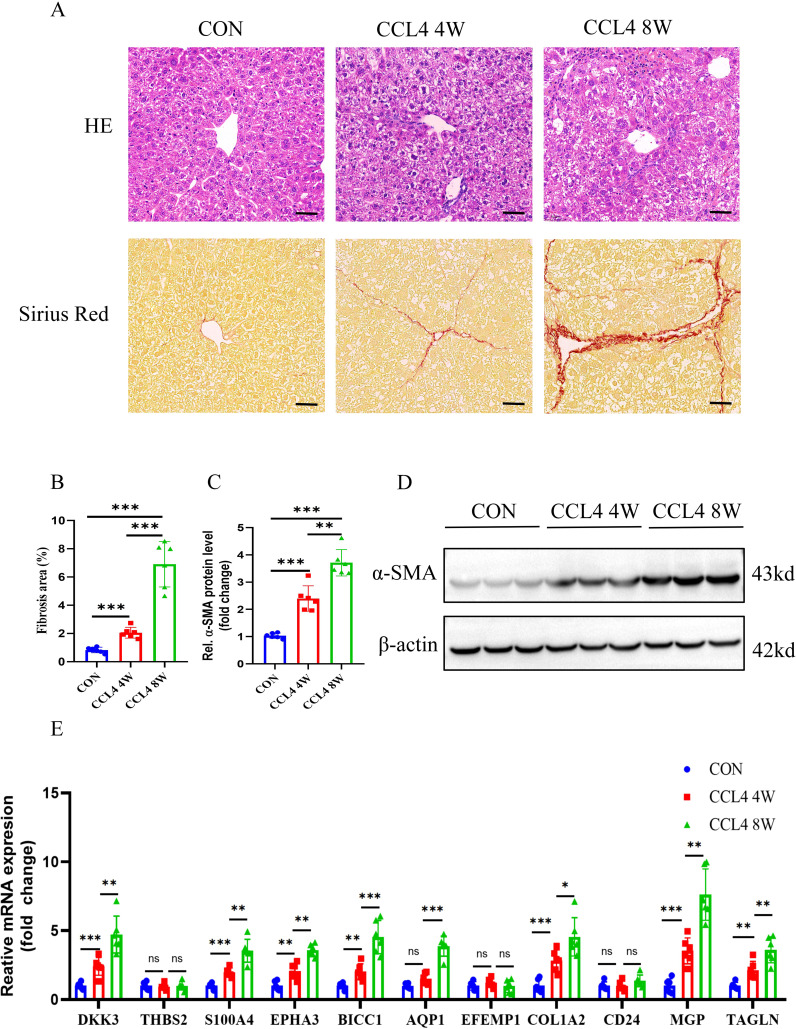
Animal experiment. **(A,B)** H&E and Sirius red staining of liver sections from mice treated on control or CCL4 (4 or 8weeks). Scale bar: 50 mm. **(B)** Semiquantitative analysis of fibrosis area. **(C)** Semiquantitative measurement for the protein levels in **(D)** Western blot assay showing the protein expression of α-SMA in the liver of the control or CCL4-treated groups(4 or 8weeks). **(E)** Determination of the key RCD-related DEGs expression in the liver of the control or CCL4-treated groups(4 or 8weeks) by RT-PCR (n=6 per group). (**, P < 0.01; ***, P < 0.001.).

To further verify the expression of characteristic genes in the progression of MASH fibrosis, we constructed a MASH fibrosis mouse model induced by feeding a MCD diet for 4 or 8 weeks. Histological examination of liver tissue from mice using HE and Oil Red O staining, revealed that MCD diet induced obviously steatosis compared with control group, and more lipid accumulation was observed in mice fed with MCD diet for 8 weeks compared to mice fed for only 4 weeks (**Figures 11A, B**). Western blot analysis indicated that the protein level of the fibrosis marker α-SMA in the liver tissue of mice on the MCD diet for 8 weeks was significantly higher than that in mice on the MCD diet for 4 weeks (**Figures 11C, D**). To analyze the correlation between the expression of 11 characteristic genes and the progression of MASH, we measured the expression levels of these genes using qPCR. As shown in **Figure 11E**, all 11 characteristic genes were up-regulated in MCD diet-fed mice compared to the control group, with expression levels gradually increasing in correlation with the severity of steatosis and fibrosis. Given the critical role of signature genes in MASH progression, we wondered whether they also play a similar role in other fibrosis models. Consequently, we constructed a CCL4-induced mouse liver fibrosis model. Results from HE, Sirius red staining and WB demonstrated that the degree of liver fibrosis increased with prolonged CCL4 treatment (**Figures 12A-D**). Additionally, qPCR results showed that DKK3, S100A4, EPHA3, BICC1, AQP1, COL1A2, MGP and TAGLN expression were up-regulated as fibrosis progressed (**Figure 12E**). Collectively, these results reveal that these genes may be involved in the development of fibrosis and could serve as molecular targets for the diagnosis and therapeutic development of fibrosis.

### Knockdown of EPHA3 inhibits TGF-β1 mediated pro-fibrotic signaling in LX2 cells

**Figure 13:**
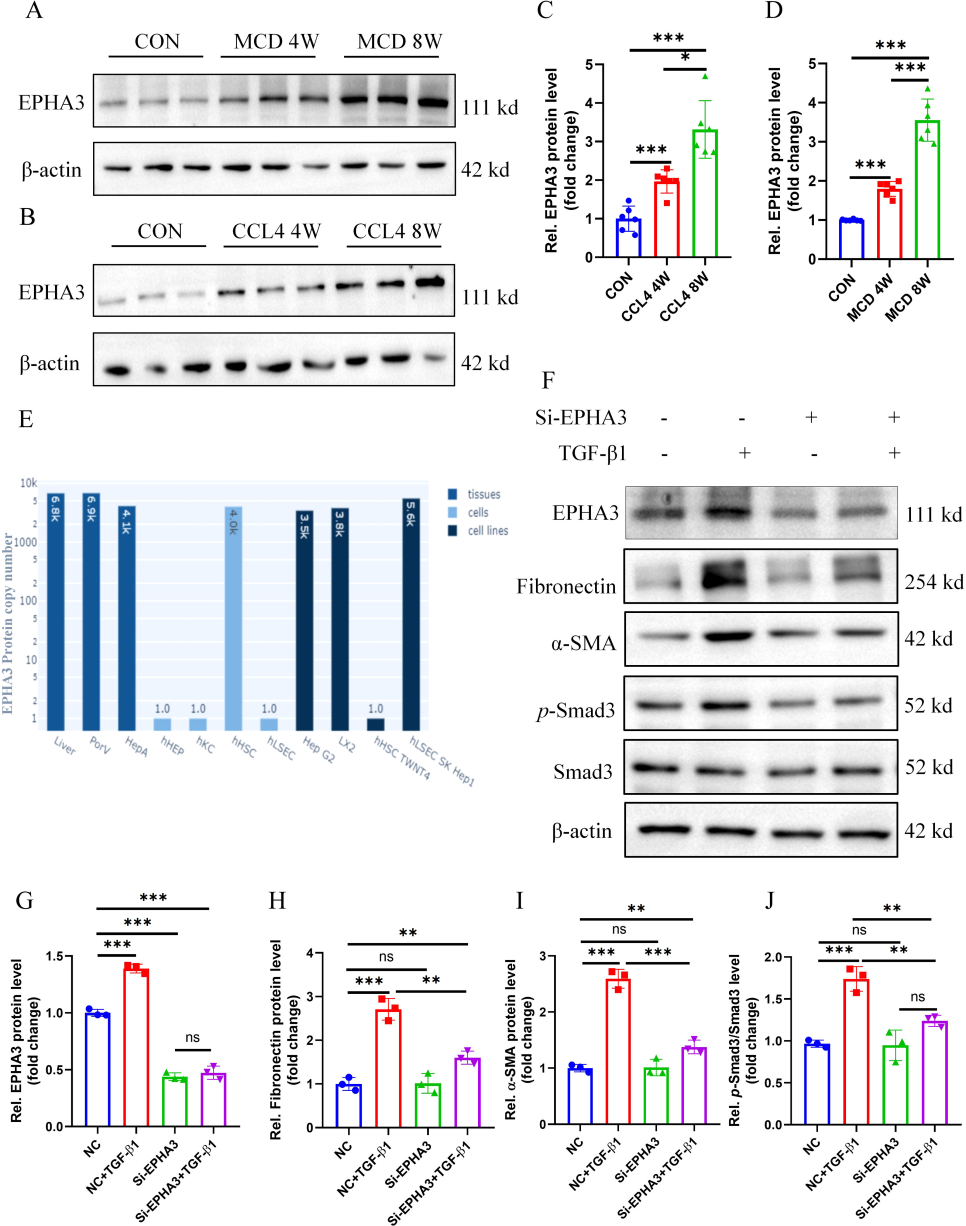
EPHA3 expression was significantly upregulated in the livers of mice with hepatic fibrosis as well as in LX2 cells treated with TGF-β1, and knockdown of EPHA3 effectively suppressed the activation of LX2 cells. **(A-D)** Western blot analysis showed the expression of EPHA3 in liver tissues of mice with MCD or CCL4-induced hepatic fibrosis, quantified using Image J software (n=6 per group). **(E)** EPHA3 expression in various cells of the liver. **(F-J)** Western blot analysis demonstrated the effect of 24h TGF-β1 (10 ng/mL) treatment on the expression of EPHA3, Fibronectin, α-SMA, and p-Smad3 in LX2 with or without si-EPHA3, quantified using Image J software (n=3 per group).(*, P < 0.05; **, P < 0.01; ***, P < 0.001; ns, no statistical signifcance.).

By screening candidate therapeutic drugs or compounds for MASH through the DGIdb database, we identified EPHA3 as the target with the highest predicted score. Therefore, we conducted a preliminary investigation on the role of EPHA3 in liver fibrosis. First, we validated the protein expression of EPHA3 in two mouse models of hepatic fibrosis, and Western blotting results showed that increased EPHA3 protein expression correlated with the degree of hepatic fibrosis (**Figures 13A-D**). In addition, we accessed the Human Liver Protein Database (http://www.liverproteome.org/) and found that EPHA3 is highly expressed in HSCs (**Figure 13E**). Subsequently, we cultured the LX2 cells *in vitro* and treated it with TGF-β1 to induce the expression of fibrosis-related genes. We observed that TGF-β activates LX2 cells and upregulates EPHA3 expression. When we utilized siRNA to knock down EPHA3 in these cells, the protein expressions of fibronectin, α-SMA and *p*-Smad3 induced by TGF-β1 were significantly reduced (**Figures 13F-J**). These findings demonstrate that knocking down EPHA3 significantly inhibited the activation of the TGF-β/Smad3 signaling pathway and the expression of its downstream fibrotic genes, thereby reducing LX2 activation.

## Discussion

MAFLD is the most prevalent chronic liver disease globally, affecting approximately 25% of the population (22). In China, MAFLD has supplanted viral hepatitis as the most common liver disorder (23). The progression of MAFLD can occur from simple steatosis to MASH, cirrhosis, and ultimately HCC (24). Research indicates that MASH has emerged as the fastest-growing cause of liver cancer (4, 21). Currently, histological evaluation remains the gold standard for diagnosing, prognosing, and monitoring treatment of MASH, however, its accuracy is highly dependent on the pathologist’s expertise (25). Consequently, there is a pressing need to identify new and reliable methods for predicting or assisting in the diagnosis of MASH. Utilizing transcriptome data, we compared the early and late stages of MASH with the aim of discovering more additional candidate diagnostic biomarkers and identifying potential therapeutic targets.

Different types of cell death play different roles in the occurrence and development of MASH (26). It is of great significance to reveal the role of RCD in the progression of MASH fibrosis.This study investigates the role of RCD in MASH for the first time and identified 11 characteristic genes based on the analysis of 10 machine learning algorithms. These signature genes are significantly up-regulated during the late stage of MASH and can accurately predict the progression of MASH fibrosis. Currently, these 11 characteristic genes are primarily associated with apoptosis, autophagy, pyroptosis, ferroptosis, copper-induced cell death, and immunogenic cell death (ICD). Increasing evidences show that apoptosis is closely related to the development of liver fibrosis. Tan and his team induced liver fibrosis in mice by injecting CCL4 and found that Fas/FasL-regulated hepatocyte apoptosis is involved in the process of liver fibrosis (27). The p53-upregulated modulator of apoptosis (PUMA) serves as a target of the Fas/FasL signaling pathway and is a crucial mediator of apoptosis (28). In the CCl4-induced liver fibrosis model, PUMA-KO knockout mice exhibited fewer apoptotic cells and lower levels of fibrosis compared to PUMA-WT wild-type mice (27). Additionally, further research has demonstrated that apoptosis directly influences HSCs by releasing apoptotic bodies or activating macrophages, which in turn leads to the activation of HSCs, thereby facilitating the progression of liver fibrosis (29). Recent studies have demonstrated that autophagic vacuoles and LC3-II levels are significantly elevated in models of liver injury (30, 31). Autophagy leads to facilitates lipophagy by mediating lipid degradation, which subsequently promotes the mobilization of lipid droplets and mitochondrial β-oxidation, thereby supplying energy for the activation of HSCs. Blocking autophagy in hematopoietic stem cells using 3-methyladenine, doxazosin or specific siRNA targeting ATG5 and ATG7 resulted in reduced activation of HSCs and attenuation of liver fibrosis (32, 33). Pyroptosis plays a significant role in the progression of liver fibrosis (34). Research has demonstrated that the activation of the NLRP3 inflammasome in mouse hepatocytes can induce both pyroptosis and liver fibrosis. Conversely, the inhibition of caspase-1 and gasdermin D (GSDMD) can suppress pyroptosis and mitigate the progression of liver fibrosis (35). In a cohort study, caspase-1 was detected in the serum of patients with MASH, and its levels were positively correlated with the severity of the disease (36). Additionally, research has demonstrated that mice overexpressing NLRP3 can spontaneously develop liver fibrosis (37). Another study found that caspase-11 levels were upregulated in the livers of MASH-affected mice, while caspase-11 knockout mice exhibited significantly reduced levels of liver injury, fibrosis, and inflammation; furthermore, levels of caspase-11, GSDMD, and IL-1β were also decreased (38). Iron overload and lipid peroxidation are commonly observed during liver injury and liver fibrosis. Su and colleagues found that liver cell-specific TAK1 deficiency leads to an imbalance of iron ions within liver cells, resulting in ferroptosis. This process triggers oxidative stress, which subsequently contributes to liver fibrosis. Notably, treatment with ferritin-1 significantly mitigated these issues (39). Additionally, Wu and colleagues identified fibroblast growth factor 21 (FGF21) as a novel inhibitor of sideroptosis. By overexpressing FGF21 in mouse hepatocytes, they demonstrated that ferroptosis can be inhibited, thereby rescuing liver fibrosis caused by iron overload (40). Consequently, inhibiting hepatocyte ferroptosis may reduce iron overload, lipid peroxidation, and inflammatory infiltration associated with liver injury, ultimately alleviating liver fibrosis (41). Cuprosis was first discovered and named by Tvetkov’s team in 2022, representing a novel form of copper-dependent cell death (42). Extracellular Cu2+ can form complexes with specific ionophores, which facilitate its passage through the cytoplasm and into the mitochondria, where it is converted into toxic compounds under the key regulation of ferredoxin 1 (FDX1) and strong Cu1+ (43–45).Currently, there are no relevant studies examining the relationship between copper wire mesh and liver fibrosis. Immunogenic cell death(ICD) can induce the body’s own cells to release DAMP and drive inflammatory responses, thereby stimulating the recruitment and activation of numerous immune cells (8). Numerous studies have demonstrated that ICD is linked to a range of diseases, including autoimmune disorders, cancer and metabolic disorders (46). While no research has yet established a correlation between ICD and liver fibrosis, gaining a comprehensive understanding of ICD and its associated regulatory factors is essential for advancing the treatment of liver fibrosis. In recent years, a growing body of research has illustrated the extensive interactions among various regulated cell death pathways that were previously considered independent. Consequently, a significant challenge lies in achieving a deeper understanding of their interaction mechanisms and how these relate to disease.

Recent studies have shown that regulated cell death is a key immunoregulatory factor that plays an important role in the occurrence, progression, and resolution of liver fibrosis (47). The microenvironment of MASH is composed of different innate immune cells and adaptive immune cells. The degree of infiltration of most innate and adaptive immune cells in late-stage MASH is higher than that in early-stage MASH (48). This study found that the proportions of immune cells such as γδ T cells, mast cells, NKT cells, and dendritic cells in the livers of patients with advanced MASH were higher than those observed in patients with early MASH. Previous studies have reported that a high-fat diet can increase the number of γδ T cells in the liver. Furthermore, TCR delta knockout mice, which lack γδ T cells, exhibited reduced liver fibrosis after being subjected to a high-fat diet, suggesting that γδ T cells may play a role in stimulating the progression of MASH (49, 50). Additionally, studies have established that mast cells are involved in tissue fibrosis (51). Concurrently, the number of mast cells in the liver is directly correlated with the advanced stage of MASH fibrosis (52). Immune cells in the liver, including NK cells, T cells, B cells, and eosinophils, are potentially linked to various forms of regulated cell death and contribute to liver fibrosis. Studies have found that NK cells and eosinophils participate in pyroptosis and necrosis, which in turn promote liver fibrosis. This study confirmed that RCD-related signature genes are positively correlated with some immune cell infiltration in MASH, indicating that they may regulate immune activation during the progression of MASH.

In this study, we identified 11 feature genes that have potential value in predicting fibrosis progression. DKK3, a glycoprotein, has been shown to regulate Wnt/β-catenin signal transduction and promote the occurrence and development of renal fibrosis. In a mouse model of chronic kidney disease (CKD), knockout of the DKK3 gene alleviated renal fibrosis (53). Furthermore, it is well-established that the Wnt/β-catenin signaling pathway plays a significant role in liver fibrosis. At present, some researchers have verified that THBS2 and its encoded secreted protein TSP-2 serve as diagnostic markers for MASH patients (54, 55). However, in this study, it was also found that THBS2 is closely related to the infiltration of immune cells in the liver of MASH patients. THBS2 may regulate the infiltration and function of immune cells in the liver by interacting with integrin receptors on the surface of immune cells. S100A4 is expressed in various cell types, including fibroblasts, macrophages, etc. Studies have found that S100A4 induces the activation of HSCs through c-Myb and exacerbates the progression of fibrosis (56). In addition, S100A4 can bind to receptors on the surface of immune cells, influencing their activity and function. TAGLN not only regulates the formation of ECM during the fibrosis process is also closely associated with mitochondrial dysfunction. Research has demonstrated that the TAGLN blocker (iTAGLN) could significantly inhibit the accumulation of ECM and reduce ROS levels (57). These findings suggested that the TAGLN blocker may serve as an effective therapeutic approach for liver fibrosis. Furthermore, some studies have confirmed that the increased expression of AEBP1 correlates with the severity of liver fibrosis in patients with MASH. Further analysis of this study revealed that AEBP1 regulates the expression of various fibrosis-related genes, including EFEMP1 (58). Our results also indicated that EFEMP1 expression was significantly elevated in advanced MASH patients and was positively correlated with the infiltration of various immune cells. EPHA3 is a crucial receptor tyrosine kinase primarily involved in the regulation of cell adhesion and migration. It participates in these processes, as well as in angiogenesis, by binding to the ligands Ephrin-B2 and Ephrin-A5. Current evidence indicated that EPHA3 was closely associated with immune cell infiltration and the efficacy of immunotherapy in tumors such as lung cancer and bladder cancer, suggesting that EPHA3 may play a role in regulating immunogenicity and the immune microenvironment (59). Notably, studies have demonstrated that the administration of anti-EPHA3 monoclonal antibodies in mice with idiopathic pulmonary fibrosis can prevent the progression of fibrosis (60). In this study, we evaluated the expression of EPHA3 in the liver tissues of mice with fibrosis induced by MCD and CCl4. The results revealed that both EPHA3 mRNA and protein levels increased in correlation with the degree of fibrosis. To further investigate the role of EPHA3 in liver fibrosis, we accessed the Human Liver Proteome Database (http://www.liverproteome.org/) and found that EPHA3 is highly expressed in HSCs. Subsequently, knocking down EPHA3 in LX2 cells resulted in a significant reduction in LX2 activation. Therefore, targeting EPHA3 may represent an important strategy for the treatment of MASH.

Based on these 11 DEGs, we screened MASH candidate therapeutic drugs or compounds through the DGIdb database.According to the ‘interactome scoring’ ranking in the DGIdb database, we identified the monoclonal antibody Ifabotuzumab (KB004), which targets EPHA3, as having the highest predicted score. The Phase I clinical study of this drug in patients with recurrent glioblastoma multiforme (GBM) has yielded promising results (61). However, the therapeutic efficacy of this drug for MASH requires further validation through animal experiments. In addition, the DNMT inhibitor Azacitidine has shown strong antifibrotic effects in both *in vivo* and *in vitro* experiments of skin fibrosis. Its mechanism involves enhancing the expression of FOXP3 in CD4+ T cells, while in skin fibroblasts, the inhibition of DNMT leads to the upregulation of the Smad3 regulator PARP1, Wnt antagonist sFRP1, and DKK1, all of which can inhibit fibrosis (62). Currently, it has been observed that the combined treatment of Bevacizumab and AAV9-LECT2-shRNA can significantly enhance the efficacy against liver fibrosis, however, a series of experimental studies are still needed for clinical application (63). Furthermore, miR-29b has been found to significantly inhibit fibrosis, and its mimic Remlarsen (MRG-201) has the potential to prevent the formation of fibrotic scars or skin fibrosis (64). The second-phase clinical trial for keloids has been completed. miR-29b serves as a negative regulator of extracellular matrix deposition and fibrosis, playing a crucial role in various fibrotic organs. In the hepatocellular carcinoma microenvironment carbonic anhydrase (CA) is able to influence immune cell function, and CA inhibitors selectively act on tumor-infiltrating macrophages to inhibit tumor growth in mice (65). In addition, squalene epoxidase (SQLE) has been found to drive MASH progression by promoting cholesterol synthesis and accumulation, as well as binding to carbonic anhydrase 3 (CA3), and combined inhibition of SQLE and CA3 using Terbinafine and Acetazolamide significantly improved MASH in mice (66). Thus, the drugs identified through the DGIdb database demonstrate certain effects on organ fibrosis and immune regulation. However, the efficacy of these drugs in treating MASH requires further confirmation through *in vivo* and *in vitro* studies.

This study has certain limitations. Although we have successfully identified genes related to MASH progression and RCD using machine learning algorithms and confirmed their diagnostic validity, the original dataset lacks detailed clinical parameters (such as survival outcomes, treatment methods, etc.). Therefore, it is essential to conduct further prospective cohort studies to explore the correlation between these characteristic genes and MASH. Additionally, a larger sample size is necessary to verify the reproducibility of MASH typing in independent cohorts from this study and systematically evaluate the predictive value of subtypes on disease progression, as well as to explore the potential for subtype-guided personalized treatment. Finally, we only performed a simple expression verification in the mouse model of liver fibrosis and briefly examined the role of EPHA3 in LX2 cells. More experiments are needed to confirm the mechanisms of these characteristic genes in the progression of liver fibrosis.

In summary, this study identified 11 RCD-related genes that are significantly upregulated in the late stages of MASH and can predicted the progression of MASH fibrosis and may even be markers of HCC. Furthermore, these characteristic genes are closely associated with immune cell infiltration and play a critical regulatory role in the liver immune microenvironment of MASH patients. We evaluated the expression of 11 characteristic genes in the liver tissues of mice with MCD or CCl4-induced hepatic fibrosis. The results indicated that these genes may be involved in the development of hepatic fibrosis. Finally, we discovered that knocking down EPHA3 in LX2 cells significantly inhibited the activation of the TGF-β/Smad3 signaling pathway and the expression of its downstream fibrogenic genes. This study offers new strategies for the clinical diagnosis of MASH fibrosis and identifies molecular targets for drug development.

## Data Availability

The datasets analyzed for this study can be found in the Gene Expression Omnibus (GEO): GSE130970 and GSE49541.
